# Flipped classroom and technology-enhanced learning in dermatology education: evidence, implementation, and future directions

**DOI:** 10.3389/fmed.2026.1955942

**Published:** 2026-09-28

**Authors:** Meng Zhang, Ruiqi Chu, Shengni Zhang, Xing Li, Xiangxiang Ren

**Affiliations:** 1 Department of Dermatology, Affiliated Hospital of Hebei University, Baoding, China; 2 Department of General Surgery, Affiliated Hospital of Hebei University, Baoding, China

**Keywords:** active learning, artificial intelligence, blended learning, dermatology education, flipped classroom, gamification, medical education, teledermatology

## Abstract

Dermatology education faces limited curriculum time, a visually demanding specialty, and the need to develop both clinical reasoning and procedural competence. The flipped classroom (FC) delivers didactic content before class and reserves class time for active learning. This mini review synthesizes evidence on FC in dermatology education, distinguishes true FC from broader blended or active-learning approaches, and examines how newer image-based technologies such as teledermatology, gamified education, and artificial intelligence (AI)-assisted tools can complement FC. PubMed, Scopus, ERIC, and Google Scholar were searched through August 2026 for studies on FC, blended learning, or technology-enhanced learning in dermatology. Across the reviewed studies, FC and closely related blended approaches were associated with short-term gains in knowledge and satisfaction relative to traditional teaching, with particular promise when combined with case-based or problem-based learning. COVID-19 accelerated adoption and demonstrated adaptability to remote formats. However, most dermatology studies are single-institution, quasi-experimental, small, and short-term, and only two dermatology-specific studies used randomized designs. Evidence on long-term retention, patient-related outcomes, and clinical competence remains lacking, and gains in satisfaction or confidence should not be equated with objective gains in knowledge or skills. Based on the currently limited evidence, FC appears at least comparable to, and sometimes better than, traditional teaching for short-term knowledge and skills. Educators can reasonably adopt FC as part of blended curricula, while researchers should prioritize multicenter randomized trials with standardized objective outcomes, longer follow-up, and patient-level endpoints.

## 1 Introduction

The flipped classroom (FC) is a pedagogical model that delivers instructional content outside class through pre-recorded videos, readings, or online modules, and reserves class time for active learning such as case discussions, collaborative problem-solving, and hands-on practice (1–3). Over the past decade, FC has been adopted across multiple health professions disciplines. Meta-analyses consistently report moderate positive effects on knowledge outcomes compared with conventional instruction (4–6). FC is a specific subtype of blended learning characterized by the core sequence of pre-class knowledge input followed by in-class higher-order application, distinguishing it from broader blended models that mix online and face-to-face components without this structured temporal inversion. Throughout this mini review, FC is used in this narrow sense; studies of general blended learning are cited only where they inform FC design principles. Related technology-enhanced approaches, including blended learning, teledermatology, gamified education, and AI-assisted tools, are presented separately as supporting or complementary evidence and not as direct tests of FC.

Applying FC to dermatology education presents both opportunities and challenges. The visual nature of the specialty makes well-designed pre-class video resources valuable for demonstrating morphologic features, while class time can be devoted to image-based quizzing, case-based reasoning, and supervised practice of procedures (1,7,8). Learners range from medical students to residents developing subspecialty expertise, and the optimal FC design likely differs across groups (7,9). The COVID-19 pandemic accelerated FC adoption by forcing rapid transition to remote and hybrid formats (8,10–12). Other blended models such as SPOC (Small Private Online Course) and TBL (Team-Based Learning) have been integrated with FC approaches in medical education and are discussed in the sections that follow.

## 2 Methods

### 2.1 Search strategy and study selection

We searched PubMed, Scopus, ERIC, and Google Scholar. The primary search was performed in July 2026 and updated on 1 August 2026 by targeted retrieval of newly published records and by screening the reference lists of included studies and recent reviews. Search strings combined the following terms in title/abstract fields: (“flipped classroom” OR “inverted classroom”) AND (“dermatology” OR “dermatological” OR ’skin') AND (“medical education” OR “undergraduate” OR “residency” OR “resident” OR “clerkship”). Additional terms included “blended learning,” 'SPOC,' “team-based learning,” “case-based learning,” and “problem-based learning.” Searches were limited to English-language publications.

### 2.2 Eligibility criteria

Studies were included if they (1) described original research, a review, or an educational intervention relevant to dermatology education; (2) evaluated a flipped classroom, blended-learning, or active-learning approach in dermatology learners; and (3) reported at least one educational outcome. We included studies at all training levels and, because few randomized trials exist in this field, accepted both controlled and single-arm designs. Conference abstracts without a full report, editorials and opinion pieces without outcome data, and studies that described FC or blended learning only outside dermatology were excluded.

### 2.3 Classification of instructional models

Eligible interventions were classified as true FC only when the publication clearly described a two-phase sequence. This sequence consisted of pre-class content delivery (for example, videos, readings, or online modules) followed by in-class active application (for example, case discussion, quizzing, or supervised practice). Studies that combined online and face-to-face components, or that used self-study, clinical teaching, practical activities, and assessment without reporting this explicit pre-class/in-class inversion, were classified as broader blended or structured curricula and are reported separately. This distinction was applied to both the narrative synthesis and Table 1.

**Table 1:** Characteristics and instructional classification of key studies in dermatology education.

Study	Design	Population	Intervention (classification)	Comparator	Key Findings
Kliesener et al. 2024 (20)	Single-arm cohort	67 med students	4-week structured curriculum (blended)	No control	Positive feedback; interactive rated highest
Osman et al. 2025 (21)	Pre-post intervention	Final-year med students	Short FC course (true FC)	Pre-intervention baseline	Significant knowledge & confidence gains
Zeng et al. 2021 (22)	Quasi- experiment	52 clinical undergraduates	SPOC + TBL blended (blended)	Traditional lecture	Higher knowledge & engagement
Li et al. 2023 (25)	RCT	78 derm residents	Micro-video FC (true FC)	Traditional lecture	Higher theoretical knowledge and clinical practice skill scores; improved autonomous learning
Malik et al. 2020 (7)	RCT	Derm residents	FC procedural demonstration (true FC)	Traditional demonstration	Better OSATS scores for biopsy, I&D, suturing
Liu et al. 2019 (26)	Prospective multi-institution	Derm surgery trainees	Video-based FC simulation (true FC)	Standard/curriculum	Improved confidence & technical performance
Muzumdar et al./2019 (28)	Single-institution/experience	Derm residents	FC dermpath/dermoscopy curriculum (implementation study)	None	Feasible implementation; no comparative outcome data
Tassavor et al. 2021 (27)	Prospective cohort	Derm surgery trainees	Remote FC surgery curriculum (true FC)	In-person FC curriculum	Feasible remote alternative

FC, flipped classroom; OSATS, Objective Structured Assessment of Technical Skills; SPOC, Small Private Online Course; TBL, Team-Based Learning. Classification: true FC denotes studies reporting a clear pre-class content-delivery phase followed by in-class active application; blended/structured denotes studies combining online, face-to-face, or active-learning components without a clearly reported FC inversion. Kliesener et al. is classified as blended/structured because the published curriculum comprised self-study, clinical teaching, practical activities, and assessment without an explicit pre-class/in-class sequence. Muzumdar et al. is presented as an implementation feasibility study, not as comparative effectiveness evidence. Studies differ in design, outcome measures, and comparator; no formal risk-of-bias weighting was applied. Sajid et al. (24), a true FC study confined to an undergraduate neuroscience/neuropharmacology block in general medical education, is not a dermatology-specific FC study and is therefore cited only as supportive general medical education evidence and is not included in Table 1.

### 2.4 Study selection, synthesis, and limitations of the narrative approach

Titles and abstracts were screened by two authors, and potentially relevant full texts were reviewed against the eligibility criteria; disagreements were resolved by discussion with a third author. Because this is a narrative mini review, not a systematic review, we did not apply a formal risk-of-bias tool or perform meta-analytic pooling. The search may have missed relevant non-English literature, gray literature, and studies not indexed in the searched databases, and classification depended on the level of instructional detail reported in each publication. Readers should interpret the findings in light of these limitations.

Studies were organized into thematic groups (FC in dermatology; FC in medical education broadly; FC combined with other pedagogies; digital and technology-enhanced approaches; COVID-19 innovations; and curriculum design) and synthesized narratively. Within each theme, outcomes are reported separately for objective measures and subjective perceptions, with attention to learner level, delivery format, and instructional design where reported.

## 3 Theoretical and empirical foundations of FC in medical education

The FC model draws on constructivist learning theory: learners build understanding through active engagement instead of passive reception (2,3). A well-designed FC allocates pre-class work to lower-order cognitive skills (remembering, understanding) and in-class work to higher-order skills (analyzing, evaluating, creating) (13). Meta-analytic evidence is mixed but generally favorable. Across 28 comparative studies, FC produced a significant but small-to-moderate knowledge benefit (SMD 0.33, 95% CI 0.21–0.46) (5). Chen et al. reviewed 46 articles but found less conclusive evidence among nine controlled studies, with a median effect size of 0.08 (6). A 2025 meta-analysis of 127 studies reported larger knowledge effects but substantial heterogeneity and evidence of small-study effects (4). These results support a probable short-term benefit, but pooled estimates vary markedly with study design, setting, and outcome assessment.

Beyond constructivism, FC aligns closely with Kolb's experiential learning theory, in which learning develops through concrete experience, reflective observation, abstract conceptualization, and active experimentation (14). Pre-class materials support abstract conceptualization and prepare learners for higher-order application, whereas in-class cases, simulations, and supervised procedures provide concrete experience, reflection, and active experimentation. Self-determination theory offers a complementary explanation: self-paced preparation supports autonomy, structured in-class tasks and feedback support competence, and collaborative discussion supports relatedness (15). These frameworks indicate that the educational value of FC depends less on the simple relocation of lectures than on deliberate alignment between pre-class preparation and in-class experiential application, interaction, and feedback.

FC has been studied across specialties with generally positive results. In nephrology, FC combined with CBL improved examination scores (16). FC-based training enhanced advanced cardiac life support skill performance (17). In nursing education, FC with problem-based learning improved critical thinking (18), and FC remained effective in fully online formats (19). Recurring barriers include student resistance to increased workload and the faculty time required for content creation (2).

## 4 FC implementation in dermatology

### 4.1 FC in undergraduate dermatology education

Undergraduate dermatology education has been evaluated through both true FC interventions and broader structured or blended curricula. A four-week structured dermatology curriculum combined self-study, clinical teaching, practical activities, and assessment; it received positive feedback, with interactive components rated most highly (20). Because the curriculum did not clearly report a pre-class/in-class inversion, it is classified here as a structured blended curriculum, not a true FC. A short-course FC intervention improved knowledge and diagnostic confidence in a low-resource setting (21). A SPOC-TBL blended model produced higher knowledge scores and engagement than traditional lectures (22), and an interpretable machine-learning analysis identified self-directed learning and independent thinking as key influences on TBL outcomes (23). As supportive general medical education evidence, a quasi-experimental FC study of an undergraduate neuroscience/neuropharmacology block (not dermatology) found no long-term retention advantage and less favorable perceptions (24). That study is included only for context and is not part of the dermatology-specific FC studies in Table 1. These studies suggest short-term benefits in knowledge and satisfaction for both true FC and broader blended formats, although nearly all are single-institution designs with immediate post-intervention assessment.

### 4.2 FC for residency training and procedural skills

At the residency level, the evidence is methodologically stronger, anchored by two randomized controlled trials. Micro-video FC produced significantly higher theoretical knowledge and clinical practice skill scores among dermatology residents, and questionnaire responses indicated improvements in autonomous learning and other aspects of comprehensive ability (25). FC-based procedural demonstrations yielded better objective skill performance for biopsy, incision and drainage, and suturing (7). A multi-institution video-based FC simulation curriculum for dermatologic surgery improved confidence and technical performance (26), and was successfully adapted for remote delivery during the pandemic (27). A single-institution experience described the implementation of an FC approach in dermatopathology and dermoscopy resident education (28). Pre-class quizzes with active-learning strategies improved engagement in health professions education (1). Key dermatology-specific studies are summarized in Table 1. Residency-level evidence is encouraging for knowledge and procedural skill outcomes, although sample sizes remain small, no study has assessed impact on patient care, and several studies relied on locally developed tests or self-reported measures instead of validated dermatology-specific assessment tools.

## 5 FC combined with other active learning strategies

CBL has been the most frequently combined strategy with FC. In dermatology residency training, a case-based collaborative learning curriculum significantly improved knowledge and was preferred over traditional lecture-based didactics (29), and combining CBL with FC further improved critical thinking in international student cohorts (30). Systematic reviews of computer-based learning interventions report improvements in learning outcomes (31), and a scoping review confirmed PBL effectiveness for clinical reasoning development (32). In dental education, both PBL and CBL improved critical thinking, though CBL was preferred (33). The rationale is that FC supplies foundational knowledge, CBL provides clinical context, and PBL adds structured inquiry (16,32). Gamification strategies including quiz competitions and point-based systems improved engagement and knowledge retention in graduate medical education (34), and game-based e-learning was more effective than conventional instruction in a randomized controlled trial (35). An integrated modular approach produced significant knowledge gains (36).

FC can also be used during simulation-based learning. In this design, pre-class materials establish baseline knowledge, procedural steps, and safety principles, allowing class or laboratory time to be devoted to simulation, deliberate practice, and structured debriefing. This sequence operationalizes experiential learning: simulation provides concrete experience and active experimentation, while debriefing promotes reflective observation and conceptual refinement. In dermatology, a video-based FC simulation curriculum improved confidence and technical performance in dermatologic surgery and was successfully adapted for remote delivery (26,27); simulation can also support dermoscopy, morphologic recognition, and procedural decision-making when linked to objective feedback (7,37,38). Simulation should not be assumed to add value simply because it is immersive or technology-enhanced; its effectiveness depends on alignment with pre-class objectives, repeated practice, and structured feedback or debriefing.

## 6 Heterogeneity and key contradictions

The studies reviewed differ across multiple dimensions. First, learner populations vary: undergraduate studies (20–22) typically use short exposures (1–4 weeks) and assess immediate knowledge gains, whereas residency studies (25–28) involve longer training and assess both knowledge and procedural competence. FC is likely more readily implemented in residency contexts, where learners have greater baseline knowledge and self-regulation capacity, although this remains an inference, not a directly tested finding. Second, delivery format matters. In-person FC and structured curricula (7,20,21) benefit from direct instructor facilitation, whereas remote FC (27) depends on digital literacy and internet access. Pandemic-era online studies (8,10–12) showed feasibility but variable engagement. Third, instructional design modulates effect size. FC combined with case-based or problem-based learning (FC + CBL, FC + PBL) (16,18,30) has been associated with favorable outcomes in several studies, likely because these methods provide clinical reasoning scaffolds relevant to a visual specialty. Direct comparisons with pure FC remain limited.

One general medical education study diverged from much of the FC literature: undergraduate students in a flipped classroom performed no better than lecture-based peers on midterm and final examinations and rated the approach less favorably (24). Several factors likely explain this divergence. Course context, outcome measures, and student familiarity with passive instruction differed from the dermatology studies above. The comparison used existing examination grades instead of a purpose-designed retention assessment, and the design did not incorporate spaced repetition or retrieval practice. Thus, positive short-term FC results do not guarantee durable learning without deliberate reinforcement.

Adaptation to dermatology presents additional considerations. General medical education meta-analyses (4–6) aggregate studies across diverse specialties and may not capture dermatology-specific factors. Dermatology relies on visual pattern recognition and morphologic description. FC effectiveness in dermatology likely depends more on the quality of visual pre-class material than in text-heavy specialties. However, the premise that visual specialties differ from text-heavy specialties in FC effectiveness is plausible but empirically untested; no cited study directly compares FC outcomes across visual versus non-visual specialties. The applicability of general FC meta-analytic conclusions to dermatology remains an extrapolation. No study has examined whether FC-trained dermatology learners achieve better clinical outcomes than traditionally trained peers.

## 7 Digital and technology-enhanced approaches

Digital technologies have long supplemented dermatology education in ways that parallel or complement FC. Early e-learning programs with online lectures and image banks improved test scores (39), and interactive case simulation systems were designed to support clinical reasoning, although they did not significantly improve final examination scores (40); these capabilities remain relevant to FC pre-class and in-class activities. Social media platforms have more recently been used to extend FC beyond the classroom, facilitating case discussions and knowledge sharing (41), and a digital dermatology toolkit improved primary care residents' comfort with describing, diagnosing, and managing common dermatologic conditions (42).

The dermatology-specific need for diverse, high-quality images intersects with broader efforts to improve representation in medical education. Skin-tone-balanced image collections have been developed to address underrepresentation of darker skin tones in traditional curricula (43). Adding images and discussion of skin disease in persons of color improved students' exposure and self-reported comfort in identifying these conditions (44), while a skin-tone-balanced curriculum reduced diagnostic performance bias (45). These resources are well suited to FC pre-class modules. Virtual patient simulations showed mixed or nonsignificant effects on knowledge but improved clinical reasoning and other skills, with low-to-modest evidence quality (37); design features associated with better outcomes included repetition until mastery, advance organizers, enhanced feedback, and explicitly contrasting cases (38). Immersive VR did not significantly improve anatomy knowledge compared with online textbooks but increased engagement and motivation (46), whereas VR and AR were as effective as tablet-based applications for anatomy learning and increased immersion (47). For dermatology FC, immersive VR could allow learners to examine three-dimensional skin lesion models and practice dermoscopy with real-time feedback (37,46), while gamified image-based quizzes with spaced repetition could improve diagnostic accuracy (34,36). Despite this potential, dermatology-specific VR/AR FC studies remain absent from the published literature.

Because dermatology is an image-based specialty, several technology-enhanced approaches have emerged alongside FC. Teledermatology, which delivers dermatologic consultation through store-and-forward or live video, has been valued as an educational tool for teaching pattern recognition and clinical reasoning to residents and medical students (48). Gamified education applies game elements to learning, and a scoping review identified game-based approaches in dermatology primarily for patient education, with potential but under-evaluated application to learner instruction (49). AI-assisted tools are advancing rapidly: a large language model used as a dermatology case narrator has been explored as a chatbot-based educational tool (50), and AI is increasingly recognized as an emerging milestone in dermatology education (51). These approaches are not competitors to FC; they share its emphasis on visual, case-based, and self-directed learning but do not by themselves guarantee the structured pre-class/in-class sequence that defines FC.

Each approach can be implemented within the two-phase FC sequence. Teledermatology cases can provide authentic pre-class clinical images or post-class consultation practice, reinforcing diagnostic reasoning (48). Gamified image-based quizzes, points, and leaderboards can structure the in-class application phase and support spaced repetition of morphologic patterns (49). AI-assisted tools can generate adaptive pre-class questions, provide automated feedback on image interpretation, or act as an interactive case narrator during self-study (50,51). Because the evidence for these technologies is largely descriptive or early-phase, they should currently be regarded as adjuncts that supplement, but do not replace, the pedagogical structure of FC, and each requires separate evaluation of its independent effect on dermatology learning outcomes.

For dermatology learners, the most relevant FC outcomes are visual pattern recognition, dermoscopic interpretation, dermatopathology correlation, procedural skill acquisition, and representative skin-of-color teaching. Assessment tools such as image-based quizzes, OSCE stations, and OSATS are better aligned with these competencies than traditional knowledge tests, but validity evidence in FC settings remains limited.

## 8 Blended learning in medical education

Blended learning, which combines face-to-face and online instruction, is conceptually related to FC and shares much of its evidence base. Meta-analyses of blended learning in health professions report higher knowledge scores than traditional instruction, with pooled effect sizes ranging from 0.81 (95% CI 0.57–1.05) across 56 interventions (52) to 1.07 (95% CI 0.85–1.28) across 56 studies (53); heterogeneity was substantial, and the estimate in Liu et al. decreased to 0.26 after trim-and-fill adjustment for publication bias (52). Digital problem-based learning was effective when online and in-class components were well-aligned (54). Digital teaching research, including FC, has grown rapidly since 2015 (55), and qualitative work found that students valued structure, high-quality online content, and tutorial support, and preferred a purposeful blend of online and face-to-face learning (56). These findings inform FC design directly: alignment between pre-class and in-class phases and careful instructional support matter more than the volume of digital content (52,54).

## 9 Impact of the COVID-19 pandemic

The COVID-19 pandemic catalyzed a rapid shift to digitally enhanced dermatology education, accelerating FC adoption across training levels. The transition from in-person to digital teaching was documented across German dermatology departments (11), and residency programs globally adopted video-based lectures, virtual grand rounds, and remote FC models (12). Curricular responses across the UK and Ireland included pre-recorded video materials and virtual case discussions, which are core FC components (8,10). Medical curricula shifted broadly toward online and FC approaches during the pandemic (57). Preclinical students valued flexible, self-paced remote learning but reported reduced participation, isolation, and loss of hands-on training (58); other surveys similarly highlighted flexibility alongside digital fatigue and engagement challenges (59,60). Single institutions rapidly implemented online classroom platforms to maintain medical education (61), and online structures helped maintain clinical education continuity when placements were suspended (62). Comparative analysis of curricular responses across nine medical schools argued that blended online components, including FC, should be retained beyond the pandemic (63).

## 10 Curriculum implications for residency training

Across these studies, several implications for curriculum design emerge. Residents consistently prefer active learning approaches over passive formats (64), supporting the theoretical rationale for FC. Online dermatology modules improved knowledge scores and were highly acceptable to learners, although the absence of a control group limited causal attribution (65). Inpatient dermatology consultation services provide exposure to complex skin disease management (66), and a structured residency preparation curriculum increased first-year residents' confidence and perceived competence in dermatology-specific clinical skills (67). The shift toward competency-based assessment in graduate medical education further aligns with the emphasis on active application and feedback that defines FC. FC can therefore be integrated into broader programmatic reform as more than an isolated teaching technique.

## 11 Discussion

### 11.1 Challenges, barriers, and study quality

Several barriers to FC adoption in dermatology persist, spanning faculty, learner, institutional, and discipline-specific domains.

Creating high-quality pre-class video content demands substantial faculty time and technical skill (25,26). In dermatology, this requirement is amplified by the need for high-resolution clinical images that accurately capture color, texture, and morphology. Image copyright and patient consent requirements add legal complexity not present in text-based specialties. The limited availability of diverse skin-tone image banks (43–45) restricts the creation of representative pre-class materials. Developing tiered pre-class content for different learner levels requires curriculum design effort that few programs can resource. Faculty development, protected time, and institutional technical support are therefore critical enabling conditions. Pre-class materials also require periodic updating to reflect evolving diagnostic criteria and treatment guidance, adding a maintenance burden beyond initial creation.

Engagement with pre-class materials is variable, and some learners fail to complete preparatory work (3,5,6). The digital divide disproportionately affects learners in resource-limited settings (21), and most FC studies have not examined whether all students can engage equally with online pre-class content.

Institutional barriers include assessment systems designed for lecture-based curricula. Standardized multiple-choice examinations often do not capture the clinical reasoning and procedural skills that FC aims to develop. This misalignment between teaching method and evaluation undermines the case for FC adoption.

The evidence base has significant quality limitations. Most published studies are single-institution, quasi-experimental designs with small samples and short-term follow-up. No dermatology FC study has reported medium- or long-term outcomes; in general medical education, a quasi-experimental comparison found no long-term retention advantage for FC (24). Among dermatology-specific FC studies, only two employed randomized designs (7,25), and neither was reported to use blinded outcome assessment. For several studies summarized in Table 1, sample size or intervention duration could not be extracted from the original publication, reflecting variable reporting standards in this literature. Because this is a narrative mini review, no formal risk-of-bias assessment or meta-analytic pooling was performed; study quality varied, and findings should be interpreted with this limitation in mind. The field lacks multicenter RCTs with sufficient power, studies using patient-level clinical outcomes as endpoints, cost-effectiveness analyses, and research conducted in resource-limited settings.

The evidence is also geographically skewed. Studies originate predominantly from Chinese academic centers with institutional technology support (22,25) and North American residency programs (26–28), with a small number from African settings where FC implementation faces connectivity and device barriers (21). No dermatology FC studies were identified from Southeast Asia, Latin America, or Eastern Europe.

An important caveat: one quasi-experimental study found no long-term retention advantage for FC and less favorable perceptions (24), suggesting that FC alone is insufficient without deliberate reinforcement.

Limitations of this review should also be acknowledged. It is a narrative mini review, not a systematic review; no protocol was registered, no formal risk-of-bias tool was applied, and no meta-analysis was performed, so the synthesis may be influenced by study selection and narrative judgment. The search was limited to English-language publications in four databases and was updated only through August 2026; relevant non-English studies, gray literature, and subsequently published work may have been missed. Classification of an intervention as true FC depended on the completeness of reporting and therefore required investigator judgment. The included dermatology studies were predominantly small, single-institution, quasi-experimental, and short-term, with only two randomized trials and no long-term or patient-level outcomes; effect estimates may therefore be unstable and susceptible to publication and small-study bias. Evidence for teledermatology, gamification, AI, and simulation within FC remains largely early-phase or indirect, and these adjuncts have not been rigorously compared with standard FC. Finally, the geographic concentration of studies in selected academic settings limits generalizability to resource-limited and diverse educational contexts. These limitations do not negate the potential value of FC, but they mean that current conclusions should be regarded as provisional.

### 11.2 Future directions

#### 11.2.1 For education researchers

Multi-center randomized trials with standardized outcome measures and follow-up of at least 12 months are needed. Given the moderate effect sizes observed in existing studies, multi-institutional consortia are likely necessary to achieve adequate statistical power. Key outcome measures should include a standardized dermatology knowledge assessment, an OSCE station for clinical reasoning, and a validated learner satisfaction instrument. Comparative effectiveness studies should test FC design variants: pure FC versus FC combined with CBL, short versus extended pre-class modules, and in-person versus hybrid delivery. Linking educational interventions to patient-level outcomes remains a critical gap that implementation science frameworks can help address. In addition, FC design should be linked to dermatology-specific competency frameworks, including milestones and entrustable professional activities. Reporting of intervention duration, completion rates, and implementation fidelity should also be standardized to support replication.

#### 11.2.2 For clinical teachers and program directors

Practical steps toward thoughtful FC adoption include the following. (1) Develop shared, peer-reviewed FC pre-class dermatology modules across institutions to address image copyright concerns through consortial licensing. (2) Create tiered pre-class curricula that differentiate core content for preclinical students, clerkship content for clinical undergraduates, and subspecialty content for residents. (3) Align assessment with FC pedagogy through image-based diagnostic assessments and OSCE stations. (4) Establish minimum technology standards and offline access options to mitigate the digital divide. (5) Integrate technology-enhanced adjuncts within the FC sequence, including teledermatology case review, gamified image-based quizzes, and AI-assisted adaptive pre-class quizzes and automated image interpretation feedback (48–51).

## 12 Conclusion

This review synthesized published evidence on FC in dermatology education and distinguished true FC interventions from broader blended or active-learning approaches. The strongest available evidence concerns objective knowledge and, in two randomized procedural studies, directly observed technical skills; these outcomes favor FC over lecture-based teaching in the short term, although the number of studies is small and follow-up has been brief. Evidence on learner satisfaction and confidence is more consistently positive, but these are subjective outcomes and should not be equated with improvements in knowledge, retention, or clinical competence. Long-term retention has not been studied in dermatology-specific FC trials, and no identified study measured patient-related outcomes or downstream clinical competence. The available evidence supports the feasibility and short-term educational value of FC, but not definitive proof of sustained or patient-level benefit. For educators, FC is a reasonable component of blended dermatology curricula alongside other active-learning strategies. Emerging image-based technologies such as teledermatology, gamified education, and AI-assisted tools can be embedded within this FC structure, but their independent effects on dermatology learning outcomes remain to be established. For researchers, multicenter randomized trials with standardized objective outcomes, longer follow-up, and patient-level endpoints remain the highest priority. Until such evidence becomes available, these conclusions should be read as provisional, not definitive, practice standards.
